# Difference in lifestyle and metabolic profile of non-alcoholic fatty liver disease with raised alanine amino-transferases between obese and non-overweight subjects

**DOI:** 10.1038/s41598-020-72306-x

**Published:** 2020-09-17

**Authors:** Mithun Sharma, Anand Kulkarni, Pramod Kumar, Vijay Bhaskar Nori, Nitin Jagtap, Rajesh Gupta, Duvurr Nageshwar Reddy, Padaki Nagaraja Rao

**Affiliations:** 1 Department of Hepatology and Liver Transplantation, Asian Institute of Gastroenterology Hospitals, Hyderabad, India; 2 Vista Diagnostics, Hyderabad, India

**Keywords:** Diseases, Gastroenterology

## Abstract

A significant proportion of patients with non-alcoholic fatty liver disease (NAFLD) in Asian sub-continent are non-overweight and may have different underlying risk factors, lifestyles and metabolic profiles. Seven hundred fifty patients of NAFLD with raised alanine-amino-transferase (ALT) were divided into non-overweight and obese group based on their body mass index (BMI). Detailed dietary and lifestyle history were obtained through questionnaires and a detailed assessment of metabolic profile and liver stiffness was done. Normal BMI (< 23 kg/m^2^) was found in 6.6% patients, of which 69.5% had raised ALT. Though the intake of dietary fat and exercise pattern were not different amongst these groups, yet the amount of aerated drinks was higher in obese subjects (12 ± 17 vs. 7 ± 7.5 *p* = 0.005). Serum low-density lipoprotein (111 ± 25.6 vs. 127.7 ± 32.7 *p* = 0.04) and insulin resistance based on HOMA-IR > 2 were significantly higher in obese group (4.1 ± 0.36 vs. 2.0 ± 0.15 *p* = 0.001). Insulin resistance and dyslipidemia were prevalent in 12% and 25% non-overweight patients respectively. Metabolic syndrome was more common in obese subjects. In addition, magnetic resonance elastography showed higher mean liver fat in the obese group with similar hepatic fibrosis. Non-overweight patients with NAFLD had lower insulin resistance and prevalence of dyslipidaemia at similar dietary and exercise pattern.

## Introduction

Non-alcoholic fatty liver disease (NAFLD) is an emerging medical problem worldwide and affects 20–34% of the Western population^1^. There is a gradual spread of this silent epidemic to South-East Asian countries, including India. The reported prevalence is between 15 and 25% in these regions^2,3^. Epidemiological studies from Indian sub-continent are rare and the exact prevalence of NAFLD is difficult to estimate. The prevalence of NAFLD with elevated alanine transferase (ALT) in India is 8.7%. Interestingly this same study found that people in India who have normal body mass index (BMI) between 18.5 and 24.9 kg/m^2^ have a two-fold increased risk of NAFLD when compared with those with BMI < 18.5 kg/m^2^^2,4^. This may be a spectrum of “lean or normal weight NAFLD” which seems different from the western fatty liver disease. NAFLD is commonly associated with obesity and metabolic syndrome. However, it can occur in non-overweight subjects also^4^.

In clinical practice, non-overweight people are least likely to be suspected of harbouring fatty liver and so are frequently missed on routine evaluation. Most of these patients are incidentally detected during routine health check-ups. Moreover, the recommended BMI cut off values for Asians for defining overweight (23–25 kg/m^2^) and obesity (> 25 kg/m^2^) are lesser than those of the western population^5^.

Data on non-overweight NAFLD is very sparse^6^. Studies from rural India have detected NAFLD in 13.2% of patients who are lean or non-overweight. Despite this, approximately 90% of such patients have dyslipidemia. In contrast, insulin resistance, which is common in obese NAFLD, was found in only 7.4% non-overweight patients^7^.

In this current study, we hypothesised that the metabolic, dietary and lifestyle profile of patients with fatty liver and raised ALT having normal BMI would be different from their obese counterparts.

This was a prospective, single-centre study of these two groups of patients where their clinical, biochemical, nutritional and lifestyle data were compared to document any significant difference.

## Methods

In this study, patients without diabetes mellitus and having non-alcoholic fatty liver were screened for eligibility in the liver clinic of our institute based on:

- Documented fatty liver on ultrasonography.
- Controlled attenuation parameter (CAP) score of > 240 on transient elastography done using Accusens 502 machine.
- Raised ALT > 40 International Units (IU).

The eligible patients were classified as non-overweight or obese based on their BMI. Those patients with NAFLD who had a BMI of < 23 kg/m^2^ were considered as lean or non-overweight while those with BMI > 25 kg/m^2^ were classified as obese (as per the Asia–Pacific guidelines which are much lower than the western standards).

NAFLD was defined as per the American Association of Study of Liver Diseases guidelines which needs evidence of steatosis either on imaging or histology. It excludes other secondary causes of fat accumulation in the liver like significant alcohol consumption, use of steatogenic medications and certain hereditary disorders like abetalipoproteinemia, lecithin-cholesterol acyltransferase deficiency, cholesterol ester storage disease and Wolman’s disease^8^. Patients already on sodium valproate, statins, anti-retroviral medications, mipomersen, lomitapide, amiodarone, methotrexate, tamoxifen or corticosteroids were excluded. Significant alcohol consumption was defined as more than 21 drinks per week in men and 14 drinks per week in women for over 2 years^8^. However, since our study subjects were not able to quantify the amount accurately, we only included those patients who have no history of alcohol consumption.

All patients at baseline underwent a complete blood count, liver function test, prothrombin time, and tests for hepatitis-B surface antigen, anti-Hepatitis-C antibody, anti-nuclear antibody, anti-liver-kidney-microsome antibody, anti-smooth muscle antibody, anti-mitochondrial antibody, immunoglobulin-G, serum iron, ferritin, ceruloplasmin and 24 h urinary copper. Patients who gave a history of use of complementary and alternative medications for any disease in the preceding six months were excluded.

Patients in whom other causes of raised ALT were ruled out and who fulfilled the definition of NAFLD were enrolled in the study. Demographic and dietary history, levels of physical activity, clinical and biochemical parameters were recorded. The BMI, waist-hip ratio and waist circumference were noted. The waist circumference was measured at the midpoint between the lower margin of the last palpable ribs and the top of the iliac crest, using a stretch‐resistant tape. Hip circumference was measured at the widest portion of the buttocks, with the tape parallel to the floor. The waist circumference divided by the hip circumference was expressed as the waist: hip ratio (as per recommendation and standardization by World Health Organization).

Dietary history was recorded in specific pre-designed questionnaires which included information regarding the type of diet (vegetarian vs non-vegetarian), amount of oil consumption per person in the family in home cooked meals, frequency of intake of fast food, aerated drinks intake which were based on patient’s recall memory. The level of physical activity was documented as sedentary, moderately active or active. Sedentary lifestyle was defined by only light day to day physical activities required for daily living. Moderately active lifestyle was defined as walking around 3–4 miles per hour or equivalent. Active lifestyle was defined by walking of more than 3 miles or equivalent. Fasting lipid profile, fasting insulin, serum homocysteine, thyroid-stimulating hormone, vitamin B-12 and vitamin D, fasting blood sugar, liver function test and magnetic resonance elastography (MRE) were done in all patients. Magnetic resonance imaging (MRI) was performed on Wipro GE OPTIMA advanced 1.5 T MRI machine (Milwaukee, USA) using the 12-element torso phased array coil. Axial sections were acquired by using 2-dimensional gradient recorded echo (GRE) sequence. An active acoustic driver was located outside the magnet room, which generated 60-Hz mechanical vibrations. This driver was connected via flexible tubing to a passive driver of 19 cm diameter to deliver the vibrations on the right side of the ribcage. Both drivers were developed by the Mayo Clinic, USA. MRE acquisition parameters included a repetition time (TR) of 50millisecs and echo time (ET) of 27millisec. Acquisition matrix size was 256 × 192 mm, the flip angle was 30°, and the section thickness was 10 mm. The field of view (FOV) was 28–40 cm and adjusted to body habitus. Four axial sections were obtained in a single breath-hold of 16 s, including the entire liver. Post-processing was performed using dedicated MR-touch software on advantage Windows 4.6 workstation. Measurement of stiffness was performed by drawing region of interest (ROI) in the non-masked portion of liver parenchyma showing well-defined, well-illuminated shear waves. The guidelines for interpretation of liver stiffness with MRE at 60 Hz were as follows^9^.

**Table:** 

< 2.5 kPa:	Normal
2.5–2.9 kPa:	Normal or inflammation
2.9–3.5 kPa:	Stage 1–2 fibrosis
3.5–4.0 kPa:	Stage 2–3 fibrosis
4.0–5.0 kPa:	Stage 3–4 fibrosis
> 5 kPa:	Stage 4 fibrosis or cirrhosis

In addition, blood samples were collected and stored for genetic assessment of Patatin-like phospholipase domain-containing protein 3 (PNPLA3) and Transmembrane 6 superfamily 2 human gene (TM6SF4) single point mutation as reported in previous studies, mostly from Asia^10–12^.

The study was approved by the Asian Institute of Gastroenterology Institutional Review Board and the Asian Institute of Gastroenterology Institutional Ethics Committee. Informed written consent was taken from all the patients. All the methods carried out in the study were in accordance with the relevant guidelines and regulations approved for the study.

### Statistical analysis

The data was analyzed using SPSS version 17. Descriptive statistics were expressed as mean ± standard deviation or median (range) for parametric or non-parametric continuous data respectively, and number (%) for categorical data. The comparison of mean between the two groups was done using paired *t* test. The categorical data were compared using Pearson’s Chi-square test or Fisher’s exact test as and when required. All statistical tests with *p* < 0.05 were considered significant.

## Results

**Figure 1:**
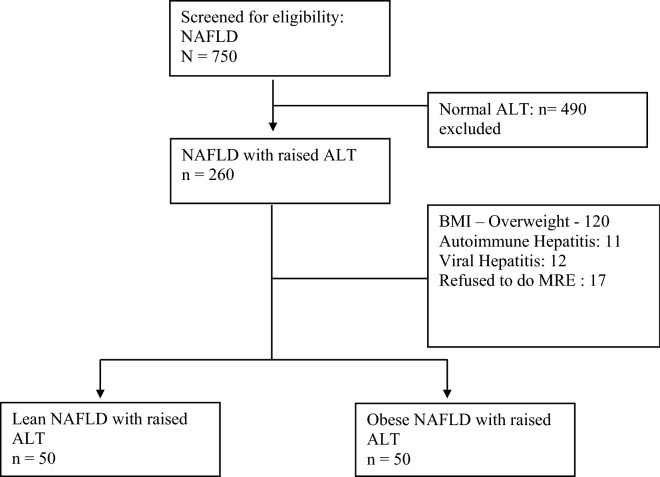
Consort diagram showing recruitment of study population and allotment to two groups. *NAFLD* non-alcoholic-fatty liver disease, *BMI* body mass index, *ALT* alanine aminotransferase, *MRE* magnetic resonance elastography, *n* number.

Seven hundred fifty patients diagnosed as NAFLD on ultrasonography were screened. Of these, 490 (65.33%) patients had normal ALT (< 40 IU) and were excluded from the study. Of the remaining 260 patients, 120/260 (46.15%) were over-weight (BMI: 23–25 kg/m^2^) and hence excluded. Eleven (4.2%) patients had positive antibodies for autoimmune hepatitis along with high immunoglobulin G levels and underwent liver biopsy, which was suggestive of autoimmune hepatitis. Twelve patients were found to have an associated viral etiology, including hepatitis B (n = 8/12), hepatitis C (n = 3) and hepatitis A (n = 1/12), and were excluded from the study. Seventeen patients refused to undergo an MRE and were not included. The final analysis had 50 patients in each arm. (Fig. 1).

When the total 750 patients of NAFLD were taken, the percentage of non-overweight patients was 6.6%. The mean age of the patients in years was 40.1 ± 9.13 and 38.1 ± 6.73 in the non-overweight and obese group, respectively. The BMI (Kg/m^2^) in the non-overweight and obese group was 21.5 ± 1.56 and 27.2 ± 2.15 respectively. As expected, the waist-hip ratio was lower in the non-overweight group (0.82 ± 0.04 vs. 0.95 ± 0.10, *p* = 0.000).

There was no difference between both groups when the amount of aerobic exercise was estimated by calculating the average time spent on walking, cycling or in the treadmill per week. The dietary history was evaluated and the average amount of oil consumed in home-cooked food was estimated as a ratio of the total amount of cooking oil in litres used per month and the number of adult family members. The average cooking oil intake per person per month was similar between the non-overweight and obese group (1.22 ± 0.46 vs. 1.39 ± 0.64, *p* = 0.287). However, there was significantly more aerated soft drinks intake/month (12 ± 1.7 vs. 17 ± 7.5 *p* = 0.005) in the obese group. Also, days of fast food intake were significantly more in the obese group (15 ± 2 vs. 20 ± 6.7 *p* = 0.004).

There was no statistically significant difference in the level of ALT (98.9 ± 34.6 vs. 96.3 ± 24.1 *p* = 0.76), serum vitamin B12 (559 ± 417.1 vs. 433 ± 185.4 *p* = 0.18) or the serum homocysteine levels (13.2 ± 4.0 vs. 14.04 + 1.80 *p* = 0.49) between the non-overweight and obese groups. Though the fasting blood sugar (90.76 ± 8.45 vs. 98.1 ± 4.2, *p* = 0.0001) was significantly different, there was only one patient whose fasting blood sugar placed them within range of a diabetes mellitus diagnosis (138mg/dl, in the non-overweight group). Mean triglyceride (154 ± 77.97 vs. 189 ± 116.9 *p* = 0.22) and HDL cholesterol (38.2 ± 6.61 vs. 39.68 ± 7.36, *p* = 0.46) also were not statistically different between the non-overweight and obese group respectively. However, the low-density lipoprotein (LDL) cholesterol levels were significantly higher in the obese group (non overweight: 111.0 ± 25.6 vs. obese: 127.7 ± 32.7 *p* = 0.04). Vitamin D level was lower in both non-overweight and obese groups(17.03 ± 7.26 vs. 18.7 ± 7.96 *p* = 0.438) without any statistical significance.

Insulin resistance was defined using a Homeostatic Model Assessment of Insulin Resistance (HOMA-IR) cut off of 2; the obese group had significantly higher mean HOMA-IR when compared to the non-overweight patients (4.1 ± 0.36 vs. 2.0 ± 0.15 *p* = 0.0001). Insulin resistance was seen in 48% of patients in the non-overweight group while it was present in 100% of patients in the obese group.

The obese group showed higher mean liver fat on MRE (19.4 ± 7.42 vs. 12.2 ± 8.69 *p* = 0.03). Fibrosis measured in kilopascals(kPa) on MRE was similar between the non-overweight and obese group (3.32 ± 1.45 vs. 3.15 ± 0.52 *p* = 0.58). However, more patients (n = 5/25, 20% vs. n = 2/25:8%) had F2-3 fibrosis in the obese group when compared with the non-overweight group. Two patients in non-overweight and four in the obese group had kPa > 6, indicating underlying cirrhosis of the liver, which was not detected on routine ultrasonography. The results are summarized in Table 1. When correlation analysis was done between the risk factors such as BMI, lipid profile, HOMA-IR and other parameters for fibrosis in the two groups, no significant positive correlation was noted.

**Table 1:** Table showing the difference of parameters between non-overweight and overweight NAFLD.

Parameter	Non-overweight n = 50 Mean ± SD	Obese n = 50 Mean ± SD	*p* value
Age in years	40.1 ± 9.13	38.1 ± 6.73	0.215
BMI (kg/m^2^)	21.5 ± 1.56	27.2 ± 2.15	0.0001**
Waist: hip ratio	0.82 ± 0.04	0.95 ± 0.10	0.001**
Alanine aminotransferase (IU)	98.9 ± 34.6	96.3 ± 24.1	0.663
Vitamin B_12_ (mg/dl)	559 ± 417.1	433 ± 185.4	0.07
Vitamin D (IU)	17.03 ± 7.26	18.74 ± 7.96	0.27
Fasting blood sugar (mg/dl)	90.76 ± 8.45	98.1 ± 4.2	0.001**
HOMA-IR	2.00 ± 0.77	4.10 ± 1.80	0.0008**
Homocysteine	13.2 ± 4.0	14.04 ± 3.52	0.27
MR fat quantification (%)	12.23 ± 8.69	19.44 ± 7.42	0.001**
MR liver stiffness (kPa)	3.32 ± 1.45	3.15 ± 0.52	0.48
LDL (mg/dl)	111 ± 25.64	127.7 ± 32.7	0.005**
HDL (mg/dl)	38.2 ± 6.61	39.68 ± 7.36	0.30
Triglycerides (mg/dl)	154.4 ± 77.97	189.32 ± 116.9	0.08
Total cholesterol (mg/dl)	160 ± 38.1	180 ± 65.2	0.06
Oil intake per person/month in litres	1.22 ± 0.46	1.39 ± 0. 64	0.13

*BMI* body mass index, *ALT* alanine aminotransaminase, *MR* magnetic resonance, *LDL* low-density lipoprotein, *HDL* high-density lipoprotein, *SD* standard deviation, *n* number, *HOMA-IR* homeostatic model assessment of insulin resistance.

**Statistically significant difference *p* < 0.005.

## Discussion

Definition of non-overweight NAFLD is still unclear with sparse data. Majority of this data have emerged from the Asian subcontinent, including India^4,7,13–16^. There is also a lack of consensus regarding the cut-off BMI to be taken for defining non-obese NAFLD. Studies from the West consider people with BMI < 25 kg/m^2^ as non-obese as opposed to BMI < 23 kg/m^2^ in Asian population^6,7,17^. A study from India has raised a pertinent question as to whether people with normal BMI or those with lower BMI < 19 kg/m^2^ should be the actual lean NAFLD^17^. However, most of the studies consider a BMI < 23 kg/m^2^ as non-obese or lean.

Considering the above BMI cut-off for Asian population, the prevalence of lean BMI in Indian patients with NAFLD varies from 11 to 32% in most studies. However, a prevalence of 89% has been reported in a study from eastern India. Most of the lean patients in this study might have had underlying undernutrition and malnourishment^4,12–19^. In our study, the percentage of NAFLD with raised ALT who were non-overweight based on BMI was 6.6%; and in this subgroup, 69.4% had persistently elevated ALT of more than twice the upper limit of normal for at least three months. The results of our study show a lower prevalence when compared to other studies from the Asia–pacific region, which have reported prevalence between 5 and 40%^17–19^. However, due to the absence of a national database, the exact prevalence of NAFLD in either non-overweight, overweight or obese patients is not clear.

Though liver biopsy is considered as the gold standard, yet due to the invasive nature of the procedure, recent guidelines have not made a liver biopsy mandatory for diagnosis of NAFLD^20^. Non-invasive tools like NAFLD fibrosis score, tissue elastography, acoustic resonance fibrosis imaging (ARFI) and MRE have emerged as alternative tools. Our study is the first study from India which have used MRE in all patients to assess fibrosis and to quantify fat in the liver. MRE has an AUROC between 92 and 100% for early detection of fibrosis. This is possibly due to the capacity of MRE to measure cross-sectional areas of hepatic parenchyma. In addition, the results are not affected by the presence of ascites or obesity^21–24^. In a recent study, which considered a BMI of < 25 kg/m^2^ to define lean NAFLD, 19.3% of 911 subjects were detected to have NAFLD on MRE. The intrahepatic triglyceride content was similar between the obese and non-obese group, and the liver stiffness was significantly lower in the non-obese group^25^. In our study, two patients in the non-obese group and four in the obese group had incidentally detected cirrhosis of liver based on a kPa value of > 6 on MRE. One of these patients later demonstrated grade I esophageal varices on endoscopy. MRE may be helpful in detecting early chronic liver disease which may be otherwise missed on routine ultrasonography.

Since metabolic syndrome and obesity is a continuous spectrum, our current study was designed to look into the two ends of the spectrum (non-overweight i.e. lean and normal BMI vs obese or high BMI) leaving the overweight NAFLD patients as a barrier to differentiate the two groups. Studies have already shown that despite being non-obese, such patients have higher visceral adiposity index and an increased risk of having components of metabolic syndrome^24^. Though previous studies have found the prevalence of metabolic syndrome to be comparatively less in non-overweight patients of NAFLD, yet the results of this study found no difference. Even though patients are lean, approximately 89% of these lean patients exhibit at least one component of metabolic syndrome^7^. In the current study, we found that only 12% of patients in the non-overweight group had one or more components of metabolic syndrome, and the most common element was dyslipidemia (25%). The components of metabolic syndrome rose to average three components (hypertension, obesity and dyslipidemia) in 50% of subjects with obesity.

In our study, we analyzed the role of diet and exercise in the development of NAFLD in subjects who had raised ALT. However, the pattern of weekly exercise in the form of aerobic activity was similar in both the groups. Thus, it can be suggested that there may be a role of some other factors like genes or gut microbiota which may influence the development of NAFLD with raised ALT in the non-overweight group. One of the limitations of the study was difficulty in documenting the level of physical activity as there was no uniform pattern of aerobic activity in the patients for most days of the week. Despite lower weight and with the expectation that exercise will not result in significant weight loss, the benefits of regular exercise even in lean subjects should not be under-stressed^26^.

The current study could not find any difference in the amount of oil (indirect marker of fat intake) used for home cooked food among the two groups. We could not analyze oil usage when the patient consumed outside/fast food. Though a questionnaire was used in the dietary assessment, the reliability of the same remains a question due to recall bias. However, there was significant aerated drink and fast food intake in the obese group. As fructose consumption has been implicated in significant deposition of hepatic fat, this may be linked with development of NAFLD in lean patients also^27^. Due to the small sample size, we were not able to demonstrate this effect. A larger study involving nutrition and genetic influence in lean patients with NAFLD is currently underway.

The most common cause of mortality in NAFLD is extra-hepatic, predominantly cardiac factors. Studies have shown an increased association of serum homocysteine levels with males, increased severity of steatosis and coronary artery disease^28,29^. In the present study, serum homocysteine levels were assessed to find out any increased risk of coronary artery disease between the two groups. However, no significant difference was observed. Vitamin B_12_, which is inversely related to the serum homocysteine level, was also similar in the two groups. The carotid intima medial thickness was however, not measured. This was a weak parameter in the study, as there may be other factors influencing the serum homocysteine levels. Whether non overweight patients with fatty liver will have the same risk of cardiac disease as their obese counterparts is something which needs to be analysed in properly designed trials.

Previous studies have found an association between serum vitamin D and NAFLD when compared to the general population^30–32^. However, when we compared the level of vitamin D between the non-overweight and obese group, no significant difference could be noted though the levels were lower in both groups. Current evidence do not show that vitamin D levels influence the fibrosis and natural history of NAFLD.

When the fasting lipid profile was compared between the two groups, dyslipidemia was found in only 25% of non-overweight compared to 60% in patients who were obese. This was in striking difference to another Asian study where 89% of lean patients had dyslipidemia^7^. In addition, our study revealed that when compared to non-overweight, the obese NAFLD had significantly higher levels of LDL cholesterol while the other parameters of lipid profile were similar. This may be in concordance with the data from Japan showing fatty liver as a significant determinant of high sd-LDL cholesterol levels independent of the presence of obesity or hyperglycemia^33^.

When most of the parameters are not significantly abnormal, the contribution of genes in the development of NAFLD should not be overlooked***. ***A non-synonymous sequence variation (rs738409) in PNPLA3 that substitutes methionine for isoleucine at residue 148(I148M) was first reported to be associated with increased hepatic triglyceride content in a genome wide screen^34^. Single nucleotide polymorphism in the PNPLA3 gene has been associated with steatosis, fibrosis and elevated transaminases in individuals with NAFLD, with variations within different regions of India. This data has been published using the same blood samples collected for genetic analysis during the study^35^. In addition to genes, gut microbiota^36^ was postulated to play an important role in the development of NAFLD and fibrosis in these patients, although it was beyond the scope of the study design.

Our study estimated the levels of vitamin B_12_ as previous studies have shown a correlation between the levels of vitamin B12 and folate with the histological severity of NAFLD^37^. No such difference could be found in this study. This may be attributed to the fact that none of the patients were on metformin which is known to lower the vitamin B_12_ levels. Moreover, inclusion of more patients with an intake of non-vegetarian diet in this study could also influence this result.

To conclude, NAFLD has been evolving both in magnitude and in concept. The mystery of progression of NAFLD to NASH and fibrosis is still poorly understood^38^. Lean or non-overweight fatty liver is an even more baffling field of hepatology, more so in Asian cohort of patients. In our current study, we concluded that non-overweight NAFLD subjects have lower insulin resistance, lower LDL cholesterol and lower hepatic fat content based on MRE. These findings, coupled with similar exercise profile and lower aerated drinks and fast food intake, led to similar ALT elevations and underlying fibrosis when compared to their obese counterparts. What lies beneath these changes and how weight loss and dietary intervention would help in non-overweight fatty liver disease is still an interesting area of research. The future research should focus on studying an integrated model which takes into account dietary, lifestyle, genetic, gut-microbiota and environmental factors and possibly make a scoring system to predict the development of NAFLD or risks for progression to Non-alcoholic steatohepatitis , fibrosis and cirrhosis.
