# Author Correction: Difference in lifestyle and metabolic profile of non-alcoholic fatty liver disease with raised alanine amino-transferases between obese and non-overweight subjects

**DOI:** 10.1038/s41598-021-87449-8

**Published:** 2021-04-08

**Authors:** Mithun Sharma, Anand Kulkarni, Pramod Kumar, Vijay Bhaskar Nori, Nitin Jagtap, Rajesh Gupta, Duvurr Nageshwar Reddy, Padaki Nagaraja Rao

**Affiliations:** 1 Department of Hepatology and Liver Transplantation, Asian Institute of Gastroenterology Hospitals, Hyderabad, India; 2 Vista Diagnostics, Hyderabad, India

Correction to: *Scientific Reports* 10.1038/s41598-020-72306-x, published online 17 September 2020

The original version of this Article contained an error.

The p value stated in the text for differences in fasting blood sugar, between non-overweight and obese groups, was incorrect. The text,

“There was no statistically significant difference in the level of ALT (98.9 ± 34.6 vs. 96.3 ± 24.1 p = 0.76), serum vitamin B12 (559 ± 417.1 vs. 433 ± 185.4 p = 0.18), fasting blood sugar (90.76 ± 8.45 vs. 98.1 ± 4.2, p = 0.12) or the serum homocysteine levels (13.2 ± 4.0 vs. 14.04 + 1.80 p = 0.49) between the non-overweight and obese groups.”

now reads,

“There was no statistically significant difference in the level of ALT (98.9 ± 34.6 vs. 96.3 ± 24.1 p = 0.76), serum vitamin B12 (559 ± 417.1 vs. 433 ± 185.4 p = 0.18) or the serum homocysteine levels (13.2 ± 4.0 vs. 14.04 + 1.80 p = 0.49) between the non-overweight and obese groups. Though the fasting blood sugar (90.76 ± 8.45 vs. 98.1 ± 4.2, p = 0.0001) was significantly different, there was only one patient whose fasting blood sugar placed them within range of a diabetes mellitus diagnosis (138 mg/dl, in the non-overweight group).”

This has been corrected in the PDF and HTML versions of the Article.

